# A Patient Friendly Corifollitropin Alfa Protocol without Routine Pituitary Suppression in Normal Responders

**DOI:** 10.1371/journal.pone.0154123

**Published:** 2016-04-21

**Authors:** Huai-Ling Wang, Hsing-Hua Lai, Tzu-Hsuan Chuang, Yu-Wei Shih, Shih-Chieh Huang, Meng-Ju Lee, Shee-Uan Chen

**Affiliations:** 1 Stork Fertility Center, Stork Ladies Clinic, Hsinchu, Taiwan (R.O.C); 2 Department of Obstetrics and Gynecology, National Taiwan University Hospital and College of Medicine, Taipei, Taiwan (R.O.C); McGill University, CANADA

## Abstract

The release of corifollitropin alfa simplifies daily injections of short-acting recombinant follicular stimulating hormone (rFSH), and its widely-used protocol involves short-acting gonadotropins supplements and a fixed GnRH antagonist regimen, largely based on follicle size. In this study, the feasibility of corifollitropin alfa without routine pituitary suppression was evaluated. A total of 288 patients were stimulated by corifollitropin alfa on cycle day 3 following with routine serum hormone monitoring and follicle scanning every other day after 5 days of initial stimulation, and a GnRH antagonist (0.25 mg) was only used prophylactically when the luteinizing hormone (LH) was ≧ 6 IU/L (over half of the definitive LH surge). The incidence of premature LH surge (≧ 10 IU/L) was 2.4% (7/288) before the timely injection of a single GnRH antagonist, and the elevated LH level was dropped down from 11.9 IU/L to 2.2 IU/L after the suppression. Two hundred fifty-one patients did not need any antagonist (87.2% [251/288]) throughout the whole stimulation. No adverse effects were observed regarding oocyte competency (fertilization rate: 78%; blastocyst formation rate: 64%). The live birth rate per OPU cycle after the first cryotransfer was 56.3% (161/286), and the cumulative live birth rate per OPU cycle after cyrotransfers was 69.6% (199/286). Of patients who did and did not receive GnRH antagonist during stimulation, no significant difference existed in the cumulative live birth rates (78.4% vs. 68.3%, p = 0.25). The results demonstrated that the routine GnRH antagonist administration is not required in the corifollitropin-alfa cycles using a flexible and hormone-depended antagonist regimen, while the clinical outcome is not compromised. This finding reveals that the use of a GnRH antagonist only occasionally may be needed.

## Introduction

The purpose of controlled ovarian stimulation (COS; [1]) in patients undergoing *in vitro* fertilization (IVF) is to obtain better reproductive outcomes by increasing the number of harvested oocytes, the number of successfully fertilized embryos, and the number of available embryos for transfer. With respect to more patient convenience, the conventional co-treatment COS protocols of gonadotropins (recombinant follicle stimulating hormone [rFSH] and recombinant luteinizing hormone [rLH]) with gonadotropin releasing hormone agonist (GnRHa), have been gradually replaced by the protocols of gonadotropins with GnRH antagonist to shorten the duration of stimulation [2]. The GnRH antagonist protocol requires a lower dose of gonadotropins without the need for a desensitization period, and yet provides remarkable outcomes with higher flexibility [3, 4]. With effective pituitary suppression, GnRH antagonist significantly decreases the rate of premature LH surge during stimulation to prevent early luteinization and follicular atresia [5].

The introduction of chimeric rFSH, corifollitropin alfa, by a combination of the human FSH α-subunit with the carboxy terminal peptide (CTP) β-subunit of human chorionic gonadotropin (hCG), has longer elimination time (~68 hours) and shorter time to reach the peak serum concentration (25~45 hours after injection) [6, 7]. A single dose of corifollitropin-alfa is able to replace daily injections of short-acting rFSH up to 7 days, and also achieves equal reproductive outcomes [8, 9]. Recently, a widely used protocol involves a single injection of corifollitropin alfa followed by fixed GnRH antagonist suppression and short-acting gonadotropin supplementation.

The release of corifollitropin alfa simplifies the traditional daily rFSH injections, and further encourages us to consider the possibility of simplifying the GnRH antagonist regimen in the corifollitropin alfa cycle. Previously, the administration of GnRH antagonist was largely based on follicle size [10], and the reported rates of premature LH surge during fixed GnRH antagonist administration varied greatly from 2.8% [11] to 22% [12] in the different approaches. It entailed that the follicle size may not directly reflect the serum LH level. We hypothesized that timely and flexible GnRH antagonist administration based on an appropriate cut-off value of serum LH (arbitrarily set as 6 IU/L, which is just over half value of the definitive LH surge) [13] may suppress the LH rise during rFSH stimulation.

Therefore, we investigated that combining an adequate hormone monitoring system with individualized and timely GnRH antagonist regimen in the corifollitropin alfa cycle could also prevent the premature LH surge. We retrospectively assessed the feasibility of corifollitropin alfa with a flexible and hormone-depended GnRH antagonist regimen in patients with regular menstrual cycles, normal baseline hormone levels, and normal ovarian reserve test result.

## Materials and Methods

### Study design

The study was approved by an appropriately constituted ethic committee of the National Taiwan University Hospital (Institutional Review Board Number: 201507061RINB), and the written informed consents were obtained from all participants. This study was a retrospective cohort analysis involving women with an indication for COS in IVF or oocyte donation programs between January 2013 and September 2014. All patients were recruited from the Outpatient Department of the Stork Fertility Center (Hsinchu, Taiwan), and were counseled by fertility specialists regarding the stimulation protocol design before treatment began. A single dose of corifollitropin alfa followed by short-acting gonadotropin supplements was administered. Serum hormone levels (luteinizing hormone [LH], oestrodial [E2], progesterone [P4]) and stimulated follicle size were monitored during the follicular phase. Flexible GnRH antagonist administration based on the monitoring serum LH levels was used in the patients. Every patient was treated with corifollitropin alfa in only one IVF cycle. Patients with multiple IVF cycles were not included in this study. The harvested blastocysts were cryopreserved. All of the patients went through frozen-thawed blastocyst transfer(s) in the other menstrual cycle(s) to decrease the OHSS risk and to increase the success rate by optimizing the endometrial synchronization [14].

### Study subjects

A total of 288 study patients were premenopausal women 23–45 years of age (mean age, 31.2 years) with regular menstrual cycles (25–35 days in length), and normal ovarian reserve test result (anti-mullerian hormone [AMH] ≧ 2 ng/mL, antral follicale count [AFC] ≧ 5). The follicular phase hormone levels (FSH, LH, E2, P4) were confirmed to be within normal ranges at baseline, and a transvaginal sonogram revealed no significant clinical abnormality. Patients with a history of ovarian hyperstimulation (OHSS), suspected to have polycystic ovary syndrome (PCOS), shown to be poor responders (≦3 oocytes retrieved in a previous stimulation cycle, or with an abnormal ovarian reserve test) [15] were ineligible for this study.

### Ovarian stimulation protocol

The injection of corifollitropin alfa (Elonva ^®^; MSD, New Jersey, USA) on cycle day 3 was followed with short-acting recombinant gonadotropins or a timely GnRH antagonist, depending on the monitored hormone results. In addition to the baseline hormone evaluation on day 3, the serum hormone levels [LH, E2, P4] and stimulated follicle size were determined every other day beginning on day 8 of a cycle. The administered dose of corifollitropin alfa was determined based on body weight (150 μg for > 60 kg and 100 μg for ≦ 60 kg; [16]). The supplemental injections of short-acting rFSH (Puregon^®^; MSD; or Pergoveris ^®^; Merck Sereno, Darmstadt, Germany) were ≦ 250 IU per day 7 days after initial stimulation if the triggering criteria was not met. Additional injections of rLH (Luveris ^®^; Merck Sereno) were depended on the serum LH level (75~150 IU of rLH per day for patients with serum LH < 0.6 IU/L). Only when the monitored serum LH level showed a sudden increase ≧ 6 IU/L (over half value of the definitive premature LH surge) would a GnRH antagonist (Cetrotide ^®^; Merck Sereno) be used (0.25 mg, *statim*). The injection frequency of GnRH antagonist was based on the monitoring serum LH result as well. No continuous GnRH antagonist was administered until the next hormone determinant.

Recombinant human chorionic gonadotropin (rHCG [250 μg], Ovidrel ^®^; Merck Sereno) was administered to trigger oocyte maturation when the dominant stimulated follicle reached 17 mm in diameter. GnRHa (triptorelin [0.2 mg], Decapeptyl ^®^; Ferring Pharmaceuticals, Ltd.; Copenhagen, Denmark) was used as the triggering medication instead of rHCG if the patient had over 15 follicles measuring up to 14 mm in diameter during stimulation to avoid OHSS [17]. Oocyte pick-up (OPU) was then performed 36 hours after triggering. The mature oocytes (metaphase II [MII]) were fertilized and then cultured to blastocyst stage.

### Hormone and ultrasound assessments

All of the patients underwent serum hormone determinations and transvaginal ultrasound scanning every other day commencing 5 days after stimulation. Before entering the stimulation cycle, the serum levels of AMH (ng/mL), FSH (IU/L), E2 (pg/mL), LH (IU/L), P4 (ng/mL), testosterone (ng/mL), prolactin (ng/mL), and thyroid stimulating hormone (mIU/L) were determined. All the hormone assays were performed at the Lezen Reference Laboratory (Taipei, Taiwan) using validated laboratory methods, enzyme-linked immunosorbent assays (ELISAs), and radioimmunoassays (RIAs). Transvaginal ovarian ultrasonography was performed to determine the size of the growing follicles, and these image results in combination with the hormone levels (LH, E2, P4), defined the maturation status of the inner oocyte. The serum β-hCG was measured 2 weeks after cryotransfer, and if positive, transabdominal ultrasonography was performed at 7 weeks gestation. Once the gestational sac and fetal heartbeat were detected, the patient was considered to have achieved a clinical pregnancy. After 16 weeks gestation, the patient was included in the clinical ongoing pregnancy group. The cumulative live birth rate was calculated in the end of this study.

### Power analysis of the sample size

The expected incidence of a LH rise in normal responders who were treated with a fixed antagonist protocol for COS is 3.2% (6/188), according to Albano et al. [18]. For our study population, a group sample size of 288 patients achieved 98% power (at an alpha level = 0.05) to detect a difference of 0.8% between the incidence of an expected LH rise if a fixed antagonist protocol was used and the 2.4% (7/288) observed incidence of premature LH surge using the hormone-depended GnRH antagonist protocol (z-test).

### Statistical analysis

The count data were presented as a percentage, and the continuous data as an average with standard deviation (SD). Multiple parameters in the group comparisons were compared using the Student *t*-test or Mann-Whitney U test, depending on the population distribution. Significant differences between two groups were defined as a two-sided *p*-value < 0.05. All the analyses were generated using scientific GraphPad software (Prism; GraphPad Software Inc., San Diego, CA, USA).

## Results

### Patient characteristics

The patient demographics are presented in Table 1. A total of 288 patients were included in this study, and the average age was 31.2 years (range, 23–45 years). The median body mass index (BMI) was 21.0 kg/m^2^ (range, 16.0–31.3 kg/m^2^). Because the majority of indications for treatment was oocyte donation, the average AMH level was 5.6 ng/mL and the median antral follicle count (AFC) was 13.0. All of the included patients had regular menstrual cycles, normal baseline hormone levels, and normal ovarian reserve test result. Patients who met the criteria as poor responders, and patients suspected with PCOS were excluded from this study cohort.

**Table 1:** Baseline characteristics.

Patient number	288
Demographics ^a^	
Age (years)	31.2±5.4
Body weight (kg)	54.2±3.4
BMI (kg/m^2^)	21.0±2.8
Indications ^b^	
Male factor	15.6% (45)
Endometriosis	2.1% (6)
Tubal factor	10.4% (30)
Oocyte–donating cycle	37.2% (107)
Unexplained	22.9% (66)
Other	11.8% (34)
Baseline hormone level ^a^	
AMH (ng/mL)	5.6±3.6
FSH (IU/L)	7.0±2.1
LH (IU/L)	2.0±1.2
E2 (pg/mL)	19.8±7.4
P4 (ng/mL)	0.7±0.3
Antral follicle count ^a^	13.0±4.2

BMI, body mass index; AMH, anti-mullerian hormone; FSH, follicle stimulating hormone; LH, luteinizing hormone; E2, oestradiol; P4, progesterone.

^a^ Data are presented as the mean±SD

^b^ Data are presented as the percentage of the class (case number of the class).

### Stimulation profiles

Table 2 presents the results of the administered medications, hormone variations, and stimulation characteristics. Most of the patients received short-acting gonadotropin supplements (96.5% [278/288]) due to the triggering criteria was not met on cycle day 10. It is noteworthy that 10 patients (3.5% [10/288]) did not require any supplemental gonadotropins 7 days after the initial corifollitropin-alfa stimulation. GnRH antagonist suppression was only used in patients with serum LH ≧ 6 IU/L. Thirty-seven patients (12.8%) received a single injection of GnRH antagonist (0.25 mg). There were 7 patients (2.4%) with an LH ≧ 10 IU/L, and the LH levels were suppressed from an average of 11.9 IU/L to 2.2 IU/L after the injection of a single GnRH antagonist. The other arm of GnRH antagonist recipients was with a LH between 6 and 10 IU/L (n = 30 [10.4%]). These patients had increased LH levels, and thus GnRH antagonist was administered prophylactically. The mean duration of stimulation was approximately 10.4 days, and oocyte retrieval was performed in 286 patients. The remaining 2 patients (38 and 42 years of age) cancelled the retrieval due to growth arrest of follicles. The average retrieved oocyte count per patient was 18.3.

**Table 2:** Stimulation Characteristics.

Patient number	288
Medication during stimulation	
Received corifollitropin alfa only ^b^	3.5% (10)
Received sa-gonadotropins ^b^	96.5% (278)
Average dose of sa-rFSH (IU) ^a^	370±140
Average dose of rLH (IU) ^a^	249±174
Received GnRH antagonist (≧ 6 IU/L) ^b^	12.8% (37)
Average dose of GnRH antagonist (mg)	0.25
Frequency of GnRH antagonist administration	1 time per cycle
Hormone variation during stimulation	
Incidence of LH rise (≧ 10 IU/L) ^b^	2.4% (7)
LH level before GnRH antagonist administration (IU/L) ^a^	11.9±1.4
LH level after GnRH antagonist administration (IU/L) ^a^	2.2±1.0
Retrieved oocyte count ^a^	18.3±7.4
Duration of stimulation (days) ^a^	10.4±1.2
OPU cancellation rate ^b^	0.7% (2)

sa-gonadotropins, short-acting gonadotropins; sa-rFSH, short-acting recombinant FSH; OPU, oocyte-pick-up.

^a^ Data are presented as the mean±SD

^b^ Data are presented as the percentage of the class (case number of the class).

### Clinical outcomes

The embryologic and reproductive outcomes are shown in Table 3. An average of 15 MII oocytes could be fertilized in each patient, and the fertilization rate (2 pronuclei [2PN]) was 78%. The day 3 good embryo rate was 67% (grading over grade II in the cleavage stage according to the Gardner and Schoolcraft system); the days 5 and 6 good blastocyst rate was 64% (grading over BC in the blastocyst stage according to the Gardner and Schoolcraft system). There were 7.5 good blastocysts to be vitrified per patient, and 2 patients (0.7% [2/286]) had no good blastocysts to be cryopreserved at the end of culture period. After the first embryo cryotransfer, the ongoing pregnancy rate per stimulation cycle (n = 288), per OPU cycle (n = 286), and per embryo transfer cycle (n = 284) were 55.9% (161/288), 56.3% (161/286), and 56.7% (161/284), respectively. The live birth rate per OPU cycle after the first cryotransfer was 56.3% (161/286), and the cumulative live birth rate per OPU cycle after cyrotransfers was 69.6% (199/286). The average embryo number per cryotransfer was 1.6, and the average cryotransfer cycle(s) per patient was 1.4.

**Table 3:** Embryologic and reproductive outcomes.

Patient number	286
Embryologic characteristics	
Number of MII oocytes ^a^	15.0±6.5
Maturation rate	82±13.5%
Fertilization (2PN) rate	78±14.9%
D3 good embryo (≧ grade II) rate	67±24.1%
D5/6 good blastocyst (≧BC) rate	64±22.2%
Number of vitrified blastocysts ^a^	7.5±4.1
No available blastocyst formed ^b^	0.7% (2)
Ongoing pregnancy rate per stimulation cycle (n = 288) after the first cryotransfer ^b^	55.9% (161)
Ongoing pregnancy rate per OPU cycle (n = 286) after the first cryotransfer ^b^	56.3% (161)
Ongoing pregnancy rate per transfer cycle (n = 284) after the first cryotransfer ^b^	56.7% (161)
Live birth rate per OPU cycle (n = 286) after the first cryotransfer ^b^	56.3% (161)
Cumulative reproductive outcomes per OPU cycle ^b^	
Clinical pregnancy rate	82.5% (236)
Implantation rate	51.8% (310/598)
Ongoing pregnancy rate	70.3% (201)
Live birth rate	69.6% (199)
Miscarriage rate	12.9% (37)
Average embryo number per cryotransfer ^a^	1.6±0.7
Average cryotransfer cycle(s) per patient ^a^	1.4±0.6

MII, metaphase II; 2PN, 2 pronuclei.

^a^ Data are presented as the mean±SD

^b^ Data are presented as the percentage of class (case number of the class).

### Comparisons between patients with and without GnRH antagonist

We also made comparisons between the patients who did and did not receive GnRH antagonist administration (LH ≧ and < 6 IU/L, respectively) during stimulation (Table 4). A total of 37 patients received GnRH antagonist injections (L-rFSH with GnRH antagonist), and 251 patients did not (L-rFSH without GnRH antagonist). There was no significant difference in the demographic characteristics between two groups. At the end of stimulation, the E2 level on the triggering day was significantly higher in the L-rFSH with GnRH antagonist group (3450±276 pg/mL *vs*. 2528±183 pg/mL, *p* = 0.02). The following embryologic and reproductive outcomes were similar in the two groups. Of patients who did and did not receive GnRH antagonist during stimulation, no significant difference existed in the cumulative live birth rate (78.4% vs. 68.3%, p = 0.25).

**Table 4:** Outcomes with and without GnRH antagonist administration.

	L-rFSH w/ GnRH antagonist	L-rFSH w/o GnRH antagonist	P-value*
Patient number	37	251	
Age (years) ^a^	31.6±3.9	31.1±5.6	0.62
BMI (kg/m^2^) ^a^	20.8±2.4	21.1±2.8	0.60
Baseline FSH (IU/L) ^a^	6.4±1.9	7.0±2.1	0.27
Baseline LH (IU/L) ^a^	2.5±2.1	2.0±1.1	0.14
Antral follicle count ^a^	12.8±4.5	13.0±4.1	0.84
E2 level on the triggering day (pg/mL) ^a^	3450±276	2528±183	0.02
Final retrieved oocyte count ^a^	20.1±7.5	18.1±7.4	0.09
Duration of stimulation (days) ^a^	10.1±0.9	10.4±1.0	0.16
OPU cancellation rate ^b^	0% (0)	0.8% (2)	1.00
Maturation rate	81±12.3%	82±13.7%	0.63
Fertilization (2PN) rate	79±1.4%	78±15.1%	0.48
D5/6 good (≧BC) blastocyst rate	62±17.2%	64±20.8%	0.56
No available blastocyst rate ^b^	0% (0)	0.8% (2)	1.00
Ongoing pregnancy rate per OPU cycle after the first cryotransfer ^b^	56.8% (21)	56.2% (140)	0.21
Live birth rate per OPU cycle after the first cyrotransfer ^b^	56.8% (21)	56.2% (140)	0.21
Cumulative reproductive outcomes per OPU cycle ^b^			
Implantation rate	54.3% (44/81)	51.5% (266/514)	0.78
Ongoing pregnancy rate	78.4% (29)	69.1% (172)	0.38
Live birth rate	78.4% (29)	68.3% (170)	0.25

L-rFSH w/ GnRH antagonist, with GnRH antagonist administration in the corifollitropin alfa cycle; L-rFSH w/o GnRH antagonist, without GnRH antagonist administration in this corifollitropin alfa cycle.

*P-values <0.05 are defined as statistically significant

^a^ Data are presented as the mean±SD

^b^ Data are presented as the percentage of the class (case number of the class).

### Endocrinology

**Fig 1:**
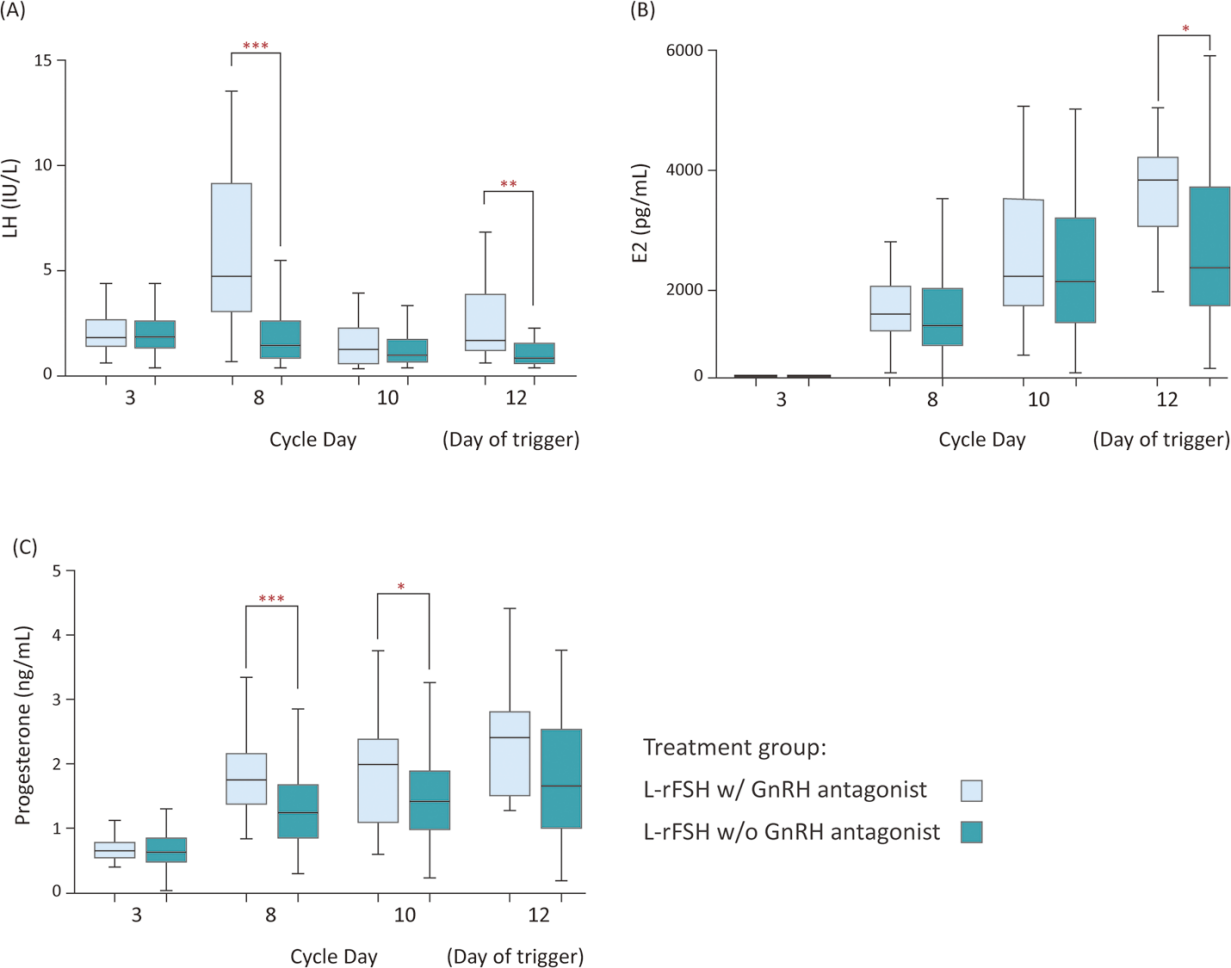
The serum hormone profiles of the patients with and without GnRH antagonist administration. L-rFSH w/ GnRH antagonist, the individuals who received GnRH antagonist in the follicular phase; L-rFSH w/o GnRH antagonist, the individuals who did not receive GnRH antagonist in the follicular phase. (A) Serum LH levels of the two groups; the LH levels on day 8 and day 12 were significantly higher in the L-rFSH with GnRH antagonist group [LH on day 8 = 6.2±3.8 IU/L *vs*. 2.0±1.7 IU/L, *p*<0.0001; LH on day 12 = 2.5±1.9 IU/L *vs*. 1.6±1.5 IU/L, *p* = 0.0047]. (B) Serum E2 levels of the two groups; the E2 level on day 12 was significantly higher in the L-rFSH with GnRH antagonist group [3450±276 pg/mL vs. 2528±183 pg/mL, *p* = 0.02]. (C) Serum P4 levels of the two groups; the P4 levels on day 8 and day 10 were significantly higher in the L-rFSH with GnRH antagonist group [P4 on day 8 = 1.8±0.5 ng/mL *vs*. 1.3±0.6 ng/mL, *p*<0.0001; P4 on day 10 = 1.9±0.9 ng/mL *vs*. 1.6±0.8 ng/mL, *p* = 0.02]. All the comparisons were using Mann-Whitney U test.

Fig 1 illustrates the serum hormone profiles of patients with and without GnRH antagonist administration (LH ≧ and < 6 IU/L, respectively). Accordingly, the LH and P4 levels of the L-rFSH with GnRH antagonist group were significantly higher than the L-rFSH without GnRH antagonist group 5 days after stimulation (LH on cycle day 8 = 6.2±3.8 IU/L *vs*. 2.0±1.7 IU/L, *p*<0.0001; P4 on cycle day 8 = 1.8±0.5 ng/mL *vs*. 1.3±0.6 ng/mL, *p*<0.0001), and thus a single injection of GnRH antagonist was used to suppress the LH elevation. Although a single dose of GnRH antagonist was administered to suppress the LH rise on day 8, the LH level increased again on day 12 in the L-rFSH with GnRH antagonist group (2.5±1.9 *vs*. 1.6±1.5, *p* = 0.0047). At this time, most patients reached the criteria of triggering, and additional suppressions to the increased LH was not required. Also, the E2 level on day 12 was statistically higher in the L-rFSH with GnRH antagonist group (3450±276 pg/mL *vs*. 2528±183 pg/mL, *p* = 0.02), also shown in Table 4.

## Discussions

The present analysis is the first cohort study to demonstrate that the use of GnRH antagonist was only occasionally required in the corifollitropin-alfa cycles combining with an appropriate serum hormone monitoring system, while the majority of patients did not need any suppression throughout the whole stimulation. The subsequent embryologic and reproductive outcomes of patients with and without GnRH antagonist administration during stimulation were similar to the end.

The primary finding was that the GnRH antagnoist was not routinely needed. The GnRH antagonist in our treatment was administered only when the serum LH level was ≧ 6 IU/L, which was half value of the definitive LH surge (LH ≧ 10 IU/L) and thus arbitrarily set as a sign of premature LH elevation, and no additional GnRH antagonist was used in advance or before the next hormone determinant. In the past, the incidence of a premature LH surge during COS was around 20% without suppression [19]. The rate of premature LH surge was greatly decreased to 2~3% by pituitary suppression with the fixed daily GnRH antagonist injection in the short-acting rFSH co-treatment [20]. With the designed hormone monitoring system and flexible GnRH antagonist regimen, the incidence of premature LH surge (LH ≧10 IU/L) was 2.4% (7/288) before the suppression, and the increased LH was suppressed from 11.9 IU/L to 2.2 IU/L after a single injection with GnRH antagonist. This percentage is much lower than that reported in the patients with fixed GnRH antagonist co-treatment in the Engage (7%) and Ensure trials (5.2%) [21], and it is more approximate to that of short-acting rFSH cycles (2.8%) [11]. Oocyte competence was not affected by the occurrence of an LH rise during stimulation; as the maturation rate, fertilization rate, good blastocyst rate, and the following reproductive outcomes were not different between patients who did and did not receive GnRH antagonist (Table 4).

Our result could support the previous data that the occurrence of an LH surge in superovulated women is controlled by the superovulation induction process itself via the interaction of E2 and gonadotropin surge-attenuating factor (GnSAF) [22]. As the acknowledged hormone feedback mechanism of human menstrual cycle, a higher concentration of GnSAF plays a negative mediator on the initial amplitude of LH pulse in the early follicular phase. With a gradual increase in E2 and decrease in GnSAF in the mid-follicular phase, the amplitude of LH pulse is strengthened. Until the periovulatory phase, the peak concentration of E2 and increased P4 easily overcome the antagonism of GnSAF and result in an LH surge with subsequent ovulation [23, 24].

In this study, the increased LH pulse (a sign of LH elevation, but not a clear LH surge) during the mid-follicular phase was temporarily suppressed by a single GnRH antagonist, but might be still intensified with the continuously increasing E2. Thus, the suppressed LH rose again on the day of triggering, as the serum E2 reached a peak. It is noteworthy that the other 251 patients (87.2%) in the study cohort did not require any GnRH antagonist because the LH remained under 6 IU/L during the whole stimulation. Although the baseline characteristics in Table 4 showed no significant difference between the patients with and without GnRH antagonist administration, the concentration of internal negative mediators to the LH pulse may be comparably lower in the patients with GnRH antagonist administration than those without. Similarly, the E2 level of patients with GnRH antagonist administration on the triggering day was relatively higher because E2 exerts positive feedback on the LH pulse.

Furthermore, a significantly lower dose of GnRH antagonist was administered in the study, and only a single injection of GnRH antagonist was applied. Compared with the previous data of single-dose regimen (3 mg) [25] and the multiple-dose regimen (0.25 mg) of GnRH antagonist, our study used a single GnRH antagonist that may suppress LH surge. However, the 0.25 mg GnRH antagonist may be unlikely effective for suppression throughout the whole stimulation for all COS populations. The antagonist had only a temporal action, which suppressed LH pulsatility only for a few hours [26]. Previous data have shown that the GnRH antagonist may not completely block the positive feedback effect of E2 in women [27]. Additional GnRH antagonist may be needed in some other COS patients if the LH rise again. In our study cohort, the LH did not rise again after the single injection of GnRH antagonist. That may result from the interaction of E2 and GnSAF during COS [22]. It is still difficult to prove that the single dose of the antagonist had any impact on the treatment outcome in the present study. This interesting issue merits further randomized clinical trials to validate the effect of single dose.

Patients with a history of poor responders, suspected PCOS, or OHSS were excluded in this analysis. Because diminished ovarian reserve was reported as a predominant risk factor for a breakthrough LH surge [28], those patients with an abnormal ovarian reserve test (AMH < 2 ng/mL) or scant AFC (< 5 follicles) were ineligible. In the study cohort, fifteen patients with over 40 years of age had normal ovarian reserve test results, and thus received corifollitropin-alfa stimulation. However, only six of these patients achieved live birth eventually. Thus their cumulative live birth rate was significantly lower (40.0%) than the totality (69.6%) [29]. In terms of the patients tolerating a higher risk of OHSS, mostly oocyte donors, GnRHa was used as a trigger. At the end, no cases of OHSS were reported in this study.

The study has the drawbacks of a retrospective analysis of data. Certainly, randomized clinical trials are needed to clarify when the use of the GnRH analogs (agonists, antagonists) is required during ovarian stimulation. In this study, LH was measured in samples taken every other day, some LH peaks might have been missed in both groups. Previous studies have shown that the LH increase may not take the form of an LH surge but that of an LH peak of short duration. A future study with detection of LH more frequently would elucidate the form of LH increase in COS. Also, the level of 6 IU/I was arbitrarily selected. It is not known whether the LH increase would have resulted in a real LH surge in these women if the antagonist had been withheld. The applicability of this cut-off value needs to be evaluated in the further studies. Before implementing the flexible GnRH antagonist regimen into the clinical application, a complete and stable hormone monitoring system is also mandatory.

In conclusion, this study demonstrated that the routine GnRH antagonist administration is not necessary in the corifollitropin-alfa cycles applying with a flexible and hormone-depended antagonist regimen, while the clinical outcome is not affected. This finding indicates that the use of a GnRH antagonist only occasionally may be needed.
